# Carotid intima-media thickness and plaques in internal carotid artery as surrogate markers of lower limb arterial lesions in Chinese patients with diabetic foot

**DOI:** 10.1590/1414-431X20198432

**Published:** 2019-07-15

**Authors:** Mei Zhang, Xiaorong Wen, Chenyun Zhou, Jing Huang, Ying He

**Affiliations:** Department of Ultrasound, West China Hospital, Sichuan University, Chengdu, Sichuan, China

**Keywords:** Diabetes mellitus, Diabetic foot, Carotid artery, Intima-media thickness, Plaque, Peripheral arterial disease

## Abstract

Peripheral arterial disease (PAD) can impair healing of diabetic foot (DF) in patients with diabetes mellitus. To determine whether carotid intima-media thickness (CIMT) can predict lower limb arterial lesions in patients with DF, this cross-sectional study enrolled patients with DF at West China Hospital (China) between January 2012 and December 2015. Ultrasonography was used to measure CIMT, assess the internal carotid arteries (ICA) for plaques, and evaluate lower limb segmental arteries for stenosis. The optimal CIMT cutoff for detecting lower limb PAD was determined by receiver operating characteristic (ROC) curve analysis. Factors associated with PAD were identified by logistic regression analyses. A total of 167 patients (mean age: 69.7±10.3 years; 102 men) were included. Patients with PAD were older and had higher levels of total cholesterol and low-density lipoprotein than patients without PAD (P<0.05). The area under the ROC curve was 0.747 (P<0.001). At the optimal CIMT cutoff of 0.71 mm, the sensitivity, specificity, positive predictive value, and negative predictive value were 79.65, 61.11, 81.08, and 58.93%, respectively. Compared with those without PAD, more patients with PAD had CIMT ≥0.71 mm (79.65 *vs* 38.89%; P<0.001) and ICA plaques (66.37*vs* 11.11%; P<0.001). Multivariate logistic regression revealed that age (odds ratio [OR]: 1.118; 95% confidence interval [95%CI]: 1.056–1.183; P<0.001), ICA plaques (OR: 13.452; 95%CI: 4.450–40.662; P<0.001), and CIMT ≥0.71 mm (OR: 2.802; 95%CI: 1.092–7.188; P=0.032) were associated with PAD.CIMT may be a surrogate marker of PAD in patients with DF.

## Introduction

Cardiovascular disease is a leading cause of death in China (1). Type-2 diabetes mellitus (T2DM) is recognized as a major risk factor for cardiovascular disease, and the overall prevalence of T2DM in China has been estimated to be 9.7%, indicating that T2DM is an important public health issue (2). Skin ulceration is a common clinical manifestation of T2DM, occurring in up to 15% of those with T2DM, and the foot is one of the most frequently affected sites (3). Diabetic foot is a common complication of T2DM that manifests as lower extremity infection, ulcer formation, and/or deep tissue damage secondary to neuropathy and peripheral vascular disease (4). Diabetic foot is an important cause of disability, amputation, and even death in people with T2DM (5). Diabetic foot complications are the most common cause of non-traumatic lower extremity amputation in underdeveloped regions and ethnic minorities (6), and the risk of lower extremity amputation is up to 46 times higher in people with T2DM than in those without T2DM (7).

Locally applied treatments, including appropriate wound care, are key to the management of neuropathic ulcers (8,9). Ulcer healing requires adequate tissue perfusion. However, T2DM is a strong risk factor for occlusive peripheral arterial disease (PAD) (10), and PAD is four times more prevalent in people with T2DM than in those without T2DM (11). People with PAD have a higher risk of all-cause, cardiovascular and coronary artery disease mortality (12). In patients with T2DM, PAD should be suspected if an ulcer fails to heal (13). PAD is present in up to 70% of patients with diabetic foot (14,15) and is associated with higher incidences of infection and disabling comorbidities (15). Given the association of PAD with poor ulcer healing and amputation, as well as cardiovascular events and premature death, it is important that PAD be diagnosed as early as possible if present in patients with diabetic foot (14). Furthermore, identifying PAD allows assessing whether revascularization surgery or angioplasty is needed to improve blood flow (16). However, a formal assessment of lower limb arterial lesions is not always made in patients with diabetic foot.

In 1986, Pignoli and colleagues reported that arterial wall intima-media thickness (IMT) could be measured non-invasively by B-mode ultrasonography (17). Carotid artery IMT (CIMT) is being increasingly used for risk stratification in individuals (18) and as an endpoint in interventional studies (19). A number of investigations have revealed the relationship between IMT and the incidence of cardiac and cerebrovascular events (20,21). Furthermore, CIMT has been reported to be a predictor of multi-territory atherosclerosis (22). However, it is not currently known whether mean CIMT and the presence of internal carotid artery plaques can predict lower limb arterial lesions in patients with diabetic foot. Therefore, in the present study, we investigated whether mean CIMT and the presence of internal carotid artery plaques could be used as markers of lower limb arterial lesions in patients with diabetic foot.

## Material and Methods

### Study participants

This cross-sectional study prospectively enrolled consecutive patients with diabetic foot who were admitted to the Diabetic Foot Care Center of West China Hospital (Sichuan University, China) between January 2012 and December 2015. The inclusion criteria were: T2DM diagnosed using internationally recognized criteria (23); a diagnosis of diabetic foot based on clinical history and examination (24); and consent for ultrasonographic measurement of CIMT and evaluation of lower limb arterial stenosis. The exclusion criteria were: previous surgery to the carotid arteries; previous surgery to the lower limbs, including amputation; ultrasonographic measurement of the CIMT failed for whatever reason; and ultrasonographic evaluation of lower limb arterial stenosis was unsuccessful, for whatever reason. The Research Ethics Committee approved this retrospective study and waived written informed consent.

### Clinical data

The following clinical data were obtained for each patient: age, gender, time since diagnosis of T2DM, glycated hemoglobin (HbA1c) level, fasting blood glucose level (FBG), plasma total cholesterol (TC), plasma triglyceride level (TG), plasma low-density lipoprotein level (LDL), systolic blood pressure (SBP), diastolic blood pressure (DBP), smoking status, and alcohol consumption. Hypertension was defined as SBP ≥140 mmHg or DBP ≥90 mm Hg or current use of antihypertensive medication to treat hypertension (25). Dyslipidemia usually refers to elevated serum high cholesterol, triglycerides, or LDL, and low HDL, commonly known as hyperlipidemia. Hyperlipidemia was defined as total cholesterol >220 mg/dL or triglycerides >170 mg/dL or >LDL 154 mg/dL. The Fontaine classification was used to classify the clinical severity of lower limb PAD as grade I: asymptomatic; II: mild-to-moderate intermittent ischemic pain and symptoms, such as intermittent claudication, and pale and cool skin; III: rest pain; or IV: ischemic ulcers or gangrene (26).

### Ultrasonographic evaluation of the carotid arteries

All ultrasound examinations were carried out by the same ultrasonographer, who had more than 15 years of experience in vascular ultrasonography. CIMT was evaluated by high-resolution B-mode ultrasonography using a Philips iU22ultrasound machine equipped with a 3–9 MHz linear array probe (Philips Healthcare, USA). Mean CIMT was automatically measured (by the IU22 system) at a plaque-free site in the far wall of the common carotid artery 1–2 cm below its bifurcation. CIMT was measured on both sides, and the higher value was selected as the value to be used in the analysis.

The bilateral internal carotid arteries were also evaluated for the presence of plaques. A plaque was defined as a maximal IMT ≥1.5 mm (27). For the analysis, each patient was classified as either having a plaque (one or more plaques detected in the internal carotid artery on at least one side) or plaque-free (no plaques detected on either side).

### Ultrasonographic evaluation of the lower limb arteries

**Figure 1.**
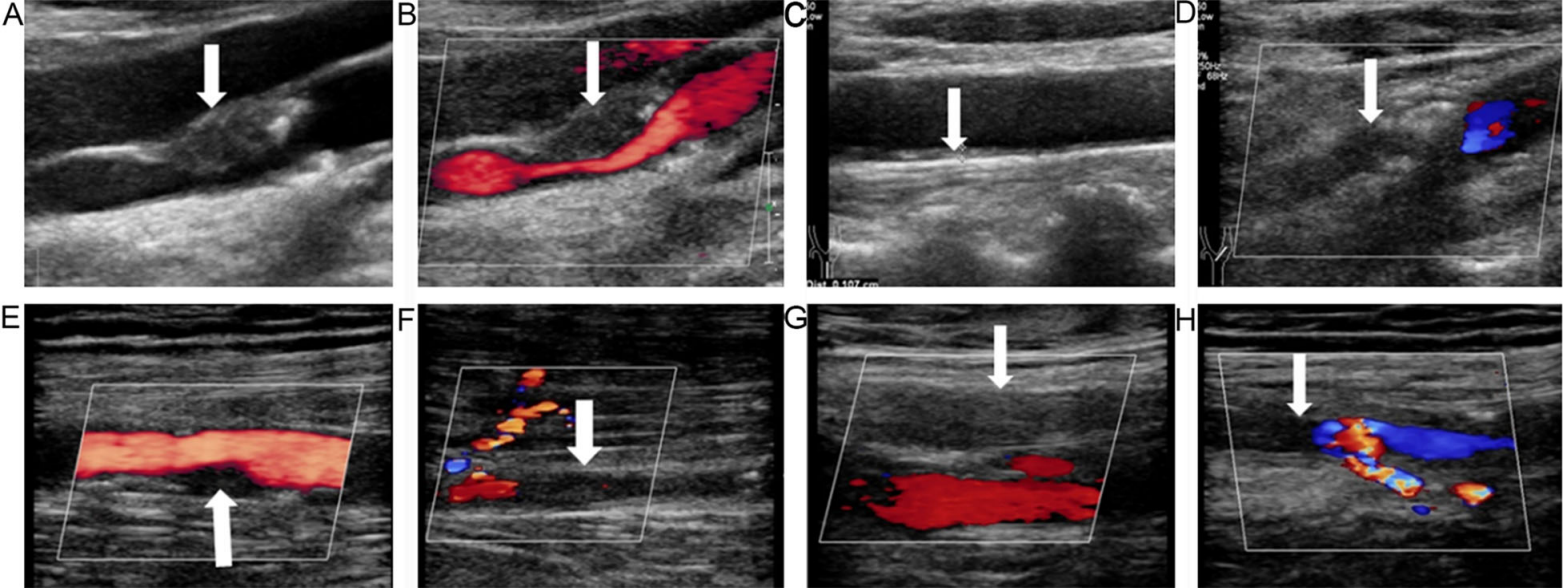
Ultrasonography of the carotid and lower limb arteries of a 74-year-old male patient with diabetic foot, peripheral arterial disease, and plaques in both internal carotid arteries. **A**, High-resolution gray-scale sonography revealed a plaque (arrow) in the front wall of the right internal carotid artery, and **C**, an increase in the intima-media thickness of the left common carotid artery (arrow; the measured value was 1.07 cm). **B**, Color Doppler ultrasound showed severe stenosis of the lumen of the right internal carotid artery (arrow; the diameter was reduced by 70–99%), **D**, indicated that the left internal carotid artery was occluded (arrow), **E**, moderate stenosis of the right superficial femoral artery (arrow; the diameter was reduced by 50–99%), **F**, segmental occlusion in the anterior tibial artery (arrow) along with a collateral artery, **G**, occlusion of the left superficial femoral artery (arrow), and **H**, occlusion of the left superficial femoral artery (arrow) accompanied by a collateral artery. The overall ultrasonic score was 10 points for the right lower limb and 9 points for the left lower limb.

Ultrasound examination of the lower limb arteries was carried out by an experienced vascular physician using the Philips IU22 ultrasound machine equipped with 5–12 MHz and 3–9 MHz probes (which had good resolution and penetration). The common femoral, superficial femoral, popliteal, anterior tibial, posterior tibial, peroneal, and dorsalis pedis arteries were evaluated (Figure 1). The degree of arterial stenosis was categorized as diameter reduced by <50%, diameter reduced by 50–99%, or complete occlusion (28). The criteria used for classifying the peripheral arterial lesions were based on duplex scanning with spectral waveform analysis: peak systolic velocity was increased by <100% relative to the adjacent proximal segment for a reduction in diameter of 0–49% and was increased by >100% relative to the adjacent proximal segment for a reduction in diameter of 50–99%; for complete occlusion, no flow was detected within the imaged arterial segment (35). An overall ultrasound-derived score for lower limb arterial disease was obtained by the piecewise integral method (29): 1 point was recorded for a 0–49% reduction in diameter, 2 points for a 50–99% reduction in diameter, and 3 points for complete occlusion. When the same artery showed narrowing in several segments, the score for the segment with the greatest narrowing was used. The arterial segmental scores in each leg were summated, and the higher ultrasonic score of the two legs was used as a measure of lower limb arterial disease. PAD was defined as any segmental artery in the lower limbs having a stenosis rate of 50% or more clinically (30).

### Statistical analysis

Statistical analysis was carried out using SPSS 22.0 (IBM, USA). Continuous variables underwent testing for normality using the Shapiro-Wilk test. Variables are reported as means±SD or median (range). Categorical variables are reported as number (%). Normally distributed continuous data were compared between groups using Student's *t*-test, while Mann-Whitney test was used for non-normally distributed data. Categorical variables were compared between groups with the χ^2^ test. Receiver operating characteristic (ROC) curve analysis with calculation of the Youden index was used to determine the optimal IMT cutoff for distinguishing between patients with and without PAD, and the area under the ROC curve (AUC), sensitivity, specificity, positive predictive value (PPV), and negative predictive value (NPV) were calculated. Univariate and multivariate logistic regression analyses were carried out to identify factors associated with PAD in patients with diabetic foot, with calculation of odds ratios (ORs) and 95% confidence intervals (95%CI). P<0.05 was taken as statistically significant.

## Results

### Clinical characteristics of the study participants

A total of 167 patients (102 men, 65 women) with diabetic foot were included in the analysis (Table 1). The age of the patients ranged from 38–89years (mean: 69.7±10.3 years). The time since diagnosis of T2DM ranged from 0–30 years (mean: 9.8±6.9 years), and HbA1c ranged from 5.7–19.9% (mean: 8.7±2.4%). According to the Fontaine classification, the severity of the lower limb PAD was graded as level I in 86 patients, level II in 17 patients, level III in 13 patients, and level IV in 51 patients.

**Table 1:** Clinical characteristics of the study participants according to the presence or absence of peripheral arterial disease (PAD).

	No PAD group (n=54)	PAD group (n=113)	Total (n=167)	P value
Age (years)	62.26±10.58	73.29±8.01	69.72±10.28	<0.001
Gender				
Female	19 (35.19%)	46 (40.71%)	65 (38.92%)	0.494
Male	35 (64.81%)	67 (59.29%)	102 (61.08%)	0.494
Duration of T2DM (years)	10 (0, 26)	10 (0.17, 30)	10 (0, 30)	0.141
Glycated hemoglobin (%)	8.7 (5.6, 18.7)	8.2 (0.8, 19.9)	8.3 (0.8, 19.9)	0.277
Fasting blood glucose (mmol/L)	8.39 (2.95, 22.60)	8.05 (2.59, 45.98)	8.29 (2.59, 45.98)	0.445
Total cholesterol (mmol/L)	3.83 (1.90, 6.51)	4.07 (1.75, 9.65)	4.00 (1.75, 9.65)	0.034
Triglycerides (mmol/L)	1.16 (0.44, 3.78)	1.29 (0.45, 6.78)	1.27 (0.44, 6.78)	0.303
Low-density lipoprotein (mmol/L)	2.07 (0.75, 4.07)	2.31 (0.58, 5.81)	2.23 (0.58, 5.81)	0.025
Systolic blood pressure (mmHg)	136.0±17.9	141.3±22.9	139.6±21.5	0.142
Diastolic blood pressure (mmHg)	79.4±9.9	76.0±12.5	77.1±11.8	0.058
Smoking				
No	31 (57.41%)	58 (51.33%)	89 (53.29%)	0.461
Yes	23 (42.59%)	55 (48.67%)	78 (46.71%)	0.461
Alcohol consumption				
No	32 (59.26%)	75 (66.37%)	107 (64.07%)	0.370
Yes	22 (40.74%)	38 (33.63%)	60 (35.93%)	0.370
Hypertension				
No	27 (50.00%)	49 (43.36%)	76 (45.51%)	0.420
Yes	27 (50.00%)	64 (56.64%)	91 (54.49%)	0.420
Hyperlipidemia				
No	45 (83.33%)	79 (69.91%)	124 (74.25%)	0.064
Yes	9 (16.67%)	34 (30.09%)	43 (25.75%)	0.064

Data are reported as means±SD, median (range), or number (percentage). T2DM: type-2 diabetes mellitus. Student's *t*-test was used for normally distributed data and Mann-Whitney test was used for non-normally distributed data. Categorical variables were compared between groups with the χ^2^ test.

Comparison of the clinical characteristics of the study participants between those with PAD (n=13) and those without PAD (n=54) are shown in Table 1. Patients with PAD were significantly older and had higher plasma levels of TC and LDL than patients without PAD (P<0.05). Patients with and without PAD showed no significant differences in gender distribution, time since diagnosis of T2DM, HbA1c, FBG, TG, SBP, DBP, prevalence of smoking, prevalence of alcohol consumption, prevalence of hypertension, or prevalence of hyperlipidemia.

### ROC curve analysis of the utility of CIMT for predicting PAD

**Figure 2.**
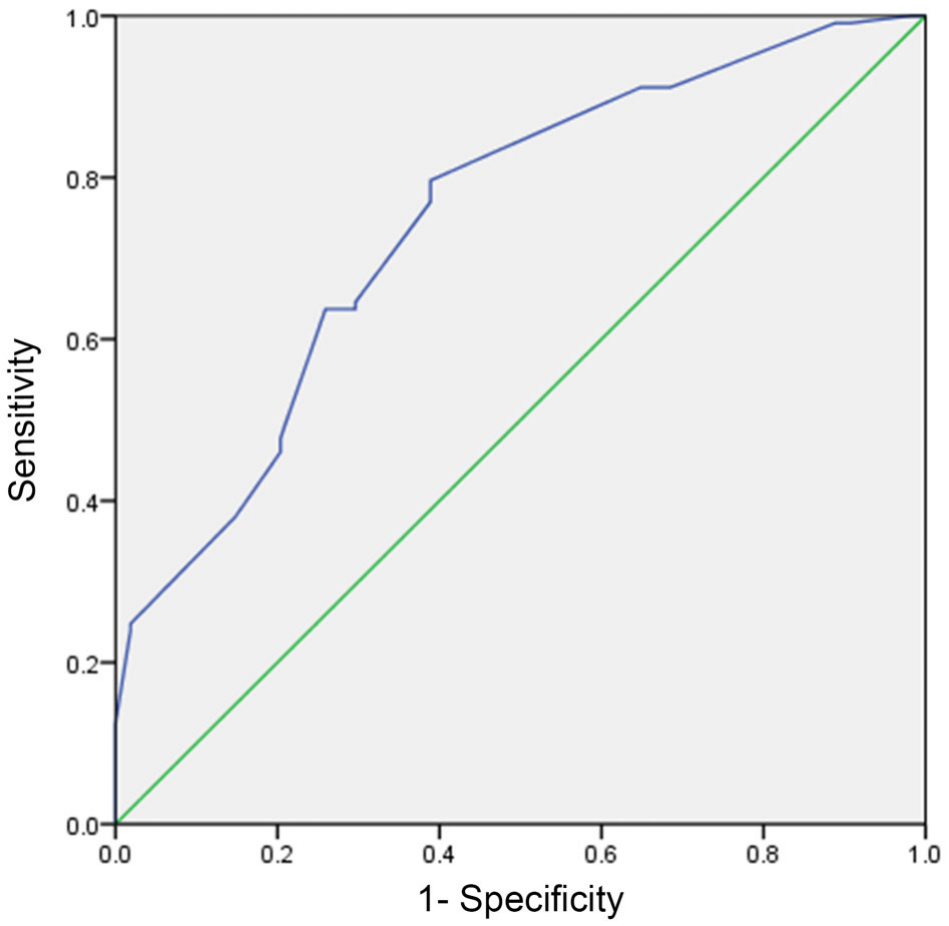
Receiver operating characteristic (ROC) curve analysis of carotid artery intima-media thickness (CIMT) for predicting lower limb peripheral arterial disease. The area under the curve was 0.747 (P<0.001), and calculation of the Youden index revealed that the optimal cutoff value for CIMT was 0.71 mm.

ROC curve analysis (Figure 2) of the ability of CIMT to predict PAD found the AUC to be 0.747 (P<0.001). Calculation of the Youden index revealed that the optimal cutoff value for CIMT was 0.71 mm. Using this cutoff value, the sensitivity, specificity, PPV, and NPV were determined to be 79.65, 61.11, 81.08, and 58.93%, respectively. A CIMT cutoff value of 0.71 mm was used for the subsequent analyses.

### CIMT and internal carotid artery plaque status of patients with and without PAD

The proportion of patients with a CIMT ≥0.71 mm was significantly higher in those with PAD (90/113, 79.65%) than in those without PAD (21/54, 38.89%; P<0.001). Furthermore, internal carotid artery plaques were present in a significantly higher proportion of patients with PAD (75/113, 66.37%) than in those without PAD (6/54, 11.11%; P<0.001).

### Univariate and multivariate logistic regression analyses of factors associated with PAD

Univariate logistic regression analysis showed that older age, higher TC, higher LDL, presence of internal carotid artery plaques, and CIMT ≥0.71 mm were significantly associated with PAD (P<0.05; see Table 2 for details). Multivariate logistic regression analysis revealed that older age (OR: 1.118; 95%CI: 1.056–1.183; P<0.001), presence of internal carotid artery plaques (OR: 13.452; 95%CI: 4.450–40.662; P<0.001), and CIMT ≥0.71 mm (OR: 2.802; 95%CI: 1.092–7.188; P=0.032) were the only factors significantly associated with PAD (Table 3).

**Table 2:** Univariate logistic regression analysis of factors associated with peripheral arterial disease.

Variable	Odds ratio	95% CI	P value
Gender	1.265	0.645, 2.479	0.494
Smoking	1.278	0.665, 2.457	0.462
Alcohol consumption	0.737	0.378, 1.438	0.371
Hypertension	1.306	0.682, 2.503	0.421
Hyperlipidemia	2.152	0.947, 4.890	0.067
Presence of internal carotid artery plaque	15.789	6.204, 40.183	<0.001
Age	1.135	1.086, 1.186	<0.001
Time since diagnosis of type-2 diabetes mellitus	1.046	0.995, 1.099	0.079
Glycated hemoglobin	0.904	0.793, 1.031	0.132
Fasting blood glucose	1.014	0.948, 1.084	0.688
Total cholesterol	1.378	1.039, 1.828	0.026
Triglycerides	1.306	0.839, 2.034	0.237
Low-density lipoprotein	1.596	1.106, 2.303	0.012
Systolic blood pressure	1.012	0.996, 1.028	0.143
Diastolic blood pressure	0.975	0.948, 1.003	0.081
Carotid intima-media thickness ≥0.71 mm	6.149	3.013, 12.551	<0.001

95%CI: 95% confidence interval.

**Table 3:** Multivariate logistic regression analysis of factors associated with peripheral arterial disease.

Variable	Odds ratio	95% CI	P value
Age	1.118	1.056, 1.183	<0.001
Gender	1.619	0.503, 5.214	0.420
Smoking	1.522	0.494, 4.694	0.464
Alcohol consumption	1.414	0.447, 4.478	0.556
Time since diagnosis of type-2 diabetes mellitus	1.001	0.935, 1.073	0.967
Glycated hemoglobin	1.018	0.841, 1.232	0.858
Hypertension	2.024	0.783, 5.230	0.145
Hyperlipidemia	1.537	0.499, 4.733	0.454
Carotid intima-media thickness ≥0.71 mm	2.802	1.092, 7.188	0.032
Presence of internal carotid artery plaque	13.452	4.450, 40.662	<0.001

95%CI: 95% confidence interval.

### Fontaine class, ultrasonic score, and number of lower extremity occluded segments between subgroups based on CIMT and internal carotid artery plaques

When the study participants were divided into two groups based on CIMT, the group with CIMT ≥0.71 mm had significantly higher values for the proportion of patients with carotid artery plaques, Fontaine class, ultrasonic score, and number of occluded segments in the lower extremities (P≤0.001; see Table 4 for details). Furthermore, when the patients were divided into two groups based on the presence or absence of internal carotid artery plaques, the group with internal carotid artery plaques had significantly higher values for Fontaine class, ultrasonic score, and number of occluded segments in the lower extremities (P<0.001; Table 5).

**Table 4:** Comparison of internal carotid artery plaque prevalence, Fontaine class, ultrasonic score, and number of lower extremity occluded segments between subgroups based on carotid intima-media thickness (CIMT).

Variable	CIMT <0.71 mm (n=56)	CIMT ≥0.71 mm (n=111)	P value
Internal carotid artery plaques			
No	39 (69.64%)	47 (42.34%)	0.001
Yes	17 (30.36%)	64 (57.66%)	0.001
Fontaine class	1 (1−5)	3 (1−5)	<0.001
Ultrasonic score	7 (7−16)	11 (7−17)	<0.001
Number of lower extremity occluded arterial segments	0 (0−4)	1 (0−5)	<0.001

Data are reported as median (range) or number (percentage). *χ* ^2^ test and Mann-Whitney test were used for analyses.

**Table 5:** Comparison of Fontaine class, ultrasonic score, and number of lower extremity occluded segments between subgroups based on the presence/absence of internal carotid artery plaques.

Variable	No plaques (n=86)	With plaques (n=81)	P value
Fontaine class	1 (1−5)	4 (1−5)	<0.001
Ultrasonic score	7 (7−17)	12 (7−16)	<0.001
Number of lower extremity occluded arterial segments	0 (0−5)	2 (0−4)	<0.001

Data are reported as median (range). Mann-Whitney test was used for analyses.

## Discussion

A notable finding of the present study was that patients with diabetic foot who had PAD were older and had higher levels of TC and LDL than those who did not have PAD. ROC curve analysis revealed that, at an optimal cutoff value for CIMT of 0.71 mm, the sensitivity, specificity, PPV, and NPV for predicting lower limb PAD were 79.65, 61.11, 81.08, and 58.93%, respectively. Importantly, a higher proportion of patients with PAD had CIMT ≥0.71 mm and ICA plaques, compared with patients without PAD. Furthermore, multivariate logistic regression revealed that older age, presence of internal carotid artery plaques, and CIMT ≥0.71 mm were associated with PAD. In addition, patients with CIMT ≥0.71 mm or internal carotid artery plaques had higher values for Fontaine class, ultrasonic score, and number of occluded segments in the lower extremities. Taken together, our novel data provide strong evidence that CIMT may be a surrogate marker of lower limb PAD in patients with diabetic foot.

Color duplex ultrasound (DUS) is an important non-invasive tool that can be used as an alternative to digital-subtraction angiography (DSA) for evaluation of lower extremity arterial disease in patients with diabetic foot. When used for the assessment of lower limb PAD, the agreement between DUS and DSA was reported to be good, irrespective of the severity of the ischemia (31). Furthermore, DUS performed better in the supragenicular arteries than in the infragenicular arteries and compared favorably with DSA in both tibial vessels, particularly in the distal part (31). An important advantage of DUS is that its cost is lower than that of both DSA and magnetic resonance angiography (MRA). The study of Lowery and colleagues (32) concluded that DUS performed extremely well compared with DSA and MRA when used as a preoperative imaging tool in the management of critical lower limb ischemia by endovascular revascularization. Since DSA is an invasive tool, it is perhaps best used for formulating a reasonable preoperative management plan rather than simply for the diagnosis of lower extremity arterial disease. In our study, ultrasonography of the lower limbs of 167 patients with diabetic foot was successful, showing good two-dimensional resolution and sensitive measurement of blood flow. Ultrasound scanning in our study permitted the segmental arteries of the lower limbs to be imaged for stenosis and occlusions, enabling the overall severity of the PAD to be graded using the piecewise integral method based on the degree of arterial stenosis. Therefore, DUS of the lower limbs represents a non-invasive and cost-effective method of assessing lower limb PAD in patients with diabetic foot.

CIMT is a surrogate marker for early atherosclerosis that can be measured relatively simply and non-invasively with high-resolution ultrasonography. CIMT is a recognized marker of coronary and cerebral artery atherosclerosis (20–22). Two methods may be used to acquire CIMT: one method involves measurements of maximal CIMT in the near and far walls of the three main segments of the carotid arteries (common carotid, bifurcation, and internal carotid); the other method involves an automated measurement of CIMT, which is restricted to the far wall of the distal common carotid artery at a plaque-free site. Computerized measurements of CIMT are superior to manual measurements in terms of precision and reproducibility, with a difference of only ∼3% between two successive automated measurements (33). Therefore, we adopted automated measurement of CIMT in the far wall of the distal common carotid artery at a plaque-free site. Our results showed that a CIMT cutoff value of 0.71 mm was able to predict lower limb PAD with a sensitivity of nearly 80% and a specificity of over 60%. Furthermore, nearly 80% of patients with PAD had CIMT ≥0.71 mm, compared with less than 40% of patients without PAD, and CIMT ≥0.71 mm was found to be an independent predictor of PAD in multivariate analysis. In addition, the group of patients with CIMT ≥0.71 mm had a higher Fontaine class, higher ultrasonic score for the lower extremities, and greater number of occluded arterial segments in the lower limbs, indicating more severe lower extremity arterial disease than patients with CIMT <0.71 mm. These findings indicate that CIMT ≥0.71 mm may be a useful surrogate marker for lower limb PAD in patients with diabetic foot. To the best of our knowledge, no previous studies have explored the relationship between CIMT and lower extremity PAD specifically in patients with diabetic foot. However, CIMT is a strong predictor of future vascular events (34), and lower extremity arterial atherosclerosis has been reported to progress more rapidly with increasing CIMT (35). Furthermore, two studies found that patients with PAD had a significantly higher CIMT than age-matched healthy controls: 0.8±0.2 mm *vs* 0.6±0.1 mm (36) and 0.85±0.35 mm *vs* 0.59±0.23 mm (37). Our finding that the optimal cutoff value for CIMT was 0.71 mm is in good agreement with these observations. The Rotterdam study determined that the OR for lower extremity arterial disease was 3.4 for people with a CIMT ≥0.89 mm (corresponding to the upper quintile) (38). Although the CIMT value in this previous study was higher than that used in ours, it should be noted that the Rotterdam study did not perform ROC curve analysis to determine an optimal cutoff value and included a study population that was not limited to patients with T2DM and diabetic foot.

There is little published data on the correlation between carotid plaques and lower limb arterial disease in patients with diabetic foot. Our study found that internal carotid artery plaques were present in a much higher proportion of patients with PAD than in those without PAD (66 *vs* 11%), and the presence of plaques in the internal carotid arteries was an independent predictor of lower limb PAD in multivariate analysis. Furthermore, patients with internal carotid artery plaques had higher values for Fontaine class, ultrasonic score, and number of occluded segments in the lower extremities. These results suggest that, in patients with diabetic foot, the existence of internal carotid artery plaques is an indicator of more serious PAD in the lower extremities. Consistent with our observations, it has been reported that carotid artery plaques are much more common in patients with PAD than in healthy controls (36). The carotid artery is a good window for observing atherosclerosis, which is a systemic disease. An increase in CIMT is considered an early marker of atherosclerosis, but plaques in the carotid arteries are not early arterial lesions. Thus, by the time that plaques emerge in the carotid arteries, it is more likely that atherosclerosis of the arteries in the lower limbs will have occurred. Indeed, it has been reported that carotid plaques are independent predictors of coronary heart disease and can improve risk prediction in older adults (39).

This study has some limitations. First, this was a single-center study with quite a small sample size, so the findings cannot be generalized. Second, this was a cross-sectional study rather than a longitudinal study, so the utility of CIMT or internal carotid artery plaques in the prediction of clinical outcomes such as foot ulcer healing was not examined. Third, other (non-ultrasonographic) methods of assessing lower extremity arterial disease, such as DSA and MRA, were not used for comparison. Additional studies are needed to further examine the clinical utility of CIMT and internal carotid artery plaques for the evaluation of lower limb PAD and prediction of ulcer healing in patients with diabetic foot.

CIMT and the presence of internal carotid artery plaques may be useful clinical markers of lower limb arterial lesions in patients with diabetic foot.
